# Chemical Control of Mosquitoes and the Pesticide Treadmill: A Case for Photosensitive Insecticides as Larvicides

**DOI:** 10.3390/insects13121093

**Published:** 2022-11-28

**Authors:** Cole J. Meier, Matthew F. Rouhier, Julián F. Hillyer

**Affiliations:** 1 Department of Biological Sciences, Vanderbilt University, Nashville, TN 37235, USA; 2 Department of Chemistry, Kenyon College, Gambier, OH 43022, USA

**Keywords:** Diptera, Culicidae, insect control, insecticide resistance, photoactive, photodynamic, pest management, reactive oxygen species

## Abstract

**Simple Summary:**

Mosquitoes transmit disease, and over the past century, mosquito control has mostly relied on chemical insecticides that target the adult life stage. We review methods of mosquito control and argue that photoactive molecules that target larvae—called photosensitive insecticides or PSIs—are an environmentally friendly addition to our mosquitocidal arsenal.

**Abstract:**

Insecticides reduce the spread of mosquito-borne disease. Over the past century, mosquito control has mostly relied on neurotoxic chemicals—such as pyrethroids, neonicotinoids, chlorinated hydrocarbons, carbamates and organophosphates—that target adults. However, their persistent use has selected for insecticide resistance. This has led to the application of progressively higher amounts of insecticides—known as the pesticide treadmill—and negative consequences for ecosystems. Comparatively less attention has been paid to larvae, even though larval death eliminates a mosquito’s potential to transmit disease and reproduce. Larvae have been targeted by source reduction, biological control, growth regulators and neurotoxins, but hurdles remain. Here, we review methods of mosquito control and argue that photoactive molecules that target larvae—called photosensitive insecticides or PSIs—are an environmentally friendly addition to our mosquitocidal arsenal. PSIs are ingested by larvae and produce reactive oxygen species (ROS) when activated by light. ROS then damage macromolecules resulting in larval death. PSIs are degraded by light, eliminating environmental accumulation. Moreover, PSIs only harm small translucent organisms, and their broad mechanism of action that relies on oxidative damage means that resistance is less likely to evolve. Therefore, PSIs are a promising alternative for controlling mosquitoes in an environmentally sustainable manner.

## 1. Introduction

More than 17% of all infectious diseases are transmitted by an invertebrate vector, and by far, the most effective vector is the mosquito [1]. The mosquito lineage (Diptera: Culicidae) originated approximately 180 million years ago, and soon thereafter, the two major mosquito subfamilies—Anophelinae and Culicinae—diverged [2,3]. Disease transmission by anophelines is exemplified by the transmission of human malaria by *Anopheles gambiae*, and disease transmission by culicines is exemplified by the transmission of dengue, yellow fever, Chikungunya and Zika by *Aedes aegypti*, and lymphatic filariasis by *Culex pipiens* [4,5,6].

The control of mosquito-borne disease relies on killing the vector, preventing vector-human contact, and using drugs to cure infection in humans [4,5,6]. These strategies have curtailed disease spread, but their efficacy is declining. Resistance against artemisinin combination therapies—the most effective antimalarial regimen—is on the rise [7,8], and although there is a vaccine that protects from yellow fever [9], there are no approved vaccines or drugs that prevent or cure infection with dengue, Zika or Chikungunya [10]. Moreover, mosquito control is hindered by the continuous evolution and spread of insecticide resistance [11,12], and while insecticide-treated nets have reduced malaria transmission [1], their effectiveness is compromised by the widespread insensitivity of mosquitoes to the pyrethroids applied to them [13]. Insecticide-treated nets also have negligible effectiveness against daytime biting and outdoor biting mosquitoes [14].

Over the past decades, mosquito control has mostly relied on chemical insecticides that target the adult life stage [4,15,16,17]. According to the World Health Organization, “during 2006–2007, about 90% of the total quantity of all classes of insecticides was reportedly used for indoor residual spraying for vector control, followed by space spraying (4%), larviciding (3.8%), treatment of mosquito nets (0.3%) and other applications (0.6%)” [16]. Over time, the percentage of insecticides used to treat mosquito nets has increased, but in every year between 2010 and 2019 the World Health Organization reports that the use of adulticides for residual spraying and space spraying has far exceeded the use of larvicides (Figure S1) [15,17]. The persistent use of these insecticides has selected for mosquito populations that are resistant to these poisons. Compared to targeting adults, little attention has been paid to curtailing mosquito populations by disrupting the larval life stage [4,12,18]. However, there are clear advantages to targeting larvae; larval death eliminates the mosquito’s entire potential to transmit disease and reproduce. Therefore, targeting the larva has benefits that are both intragenerational (fewer biting vectors within a generation) and transgenerational (population suppression across generations), and is an underutilized strategy. In the United States, the Environmental Protection Agency (EPA) and the Centers for Disease Control and Prevention advocate for integrative pest management strategies that first focus on killing larvae [19], although a recent survey of vector control programs in Florida and Texas found that 100% of responding districts target adults but only 86% target larvae [20]. In this review, we briefly summarize current chemical methods of mosquito control and then argue that photoactive molecules that target larvae—henceforth called photosensitive insecticides or PSIs—are an environmentally friendly alternative that could be used to kill mosquitoes in a manner that has minimal detrimental effects on the general ecosystem.

## 2. Targeting Mosquito Adults: Classical Chemical Insecticides and the Pesticide Treadmill

**Figure 1:**
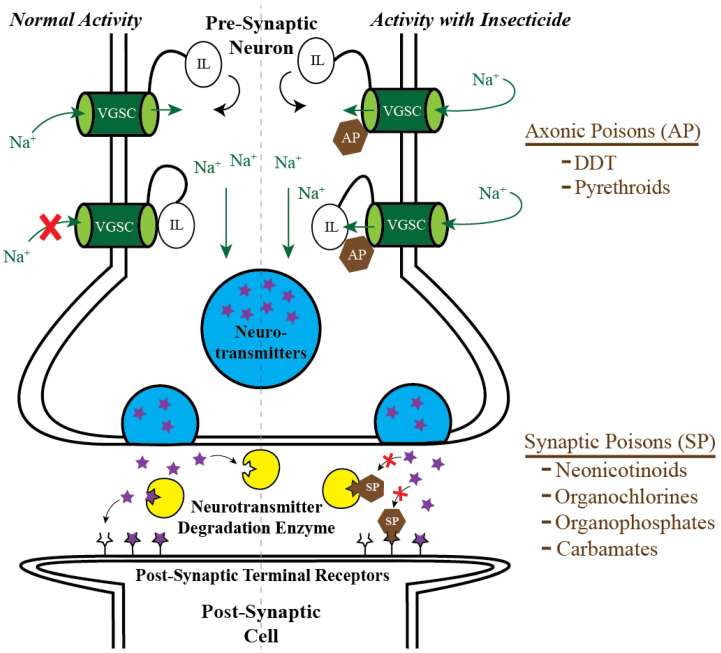
**Neurotoxic insecticides can be classified as axonic poisons or synaptic poisons.** Axonic poisons (AP) bind voltage gated sodium channels (VGSC) and their inactivation loop (IL), thereby altering the movement of sodium ions and disrupting the transmission of action potentials. Synaptic poisons (SP) either prevent enzymes from degrading neurotransmitters or interfere with the binding of neurotransmitters to their post-synaptic terminal receptors, thereby disrupting the communication between presynaptic neurons and post-synaptic cells.

Insecticides reduce the spread of mosquito-borne disease [4,21]. Most classical insecticides—such as pyrethroids, neonicotinoids, chlorinated hydrocarbons, carbamates and organophosphates—kill insects by disrupting specific neurophysiological processes [12,22,23,24,25,26]. These neurotoxic insecticides can be mechanistically sub-classified into axonic poisons and synaptic poisons (Figure 1). Axonic poisons—such as pyrethroid derivatives and chlorinated hydrocarbons like dichloro-diphenyl-trichloroethane or DDT—bind to voltage gated sodium channels along the axon of neurons and alter the kinetics of depolarization, thereby disrupting the transmission of action potentials along an axon [24,27]. Synaptic poisons—such as neonicotinoids, carbamates, organophosphates, and some chlorinated hydrocarbons—disrupt enzymes in the synaptic cleft that either degrade neurotransmitters or alter the binding of neurotransmitters to their post-synaptic receptors, thereby disrupting the proper communication between presynaptic neurons and post-synaptic cells [24,28]. The outcome for both axonic and synaptic poisons is the disruption of neural and neuromuscular communication, leading to death.

**Figure 2:**
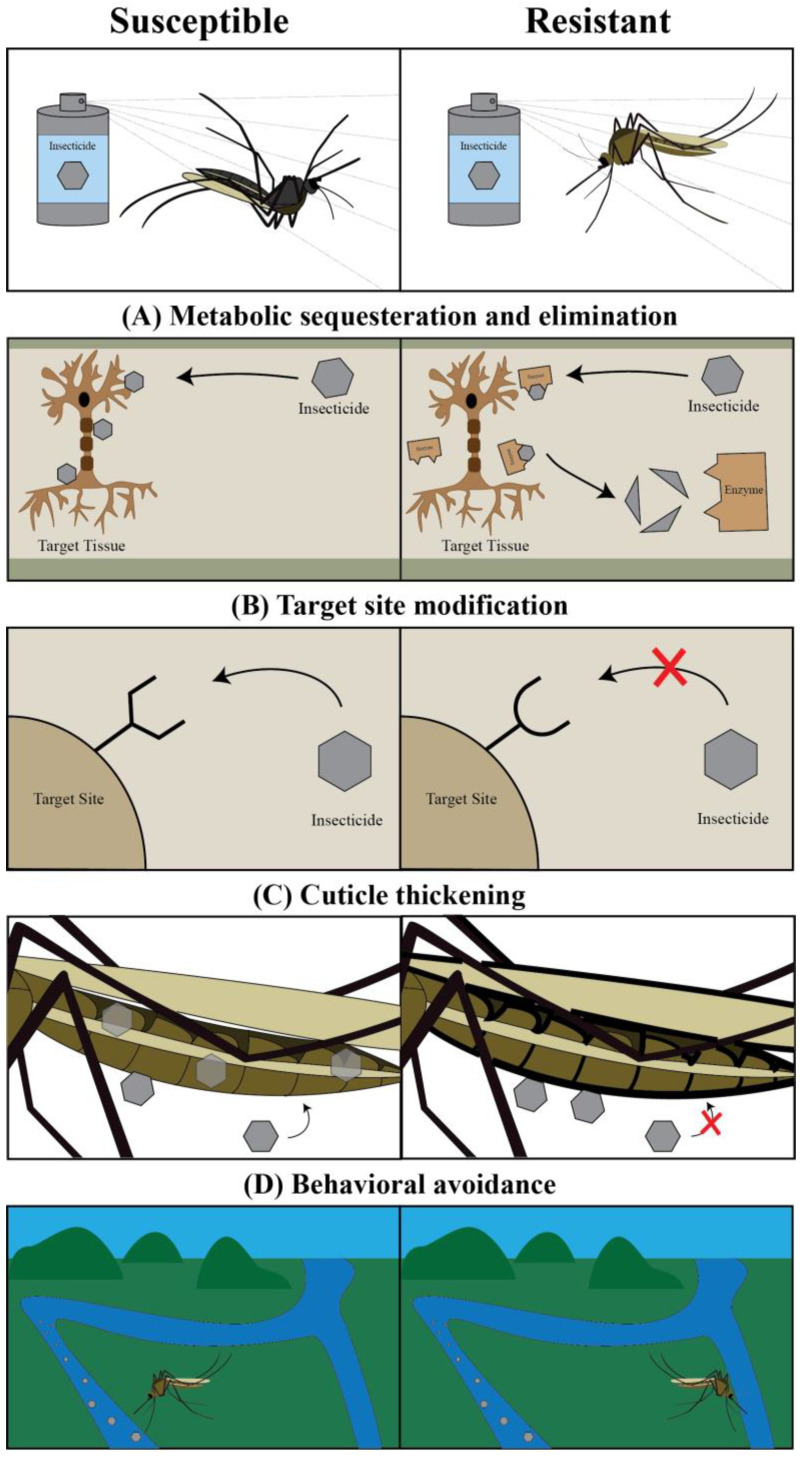
**Insects utilize four primary strategies to evolve resistance against insecticides: metabolic sequestration and elimination, target site modification, cuticle thickening, and behavioral avoidance.** (**A**) Metabolic sequestration and elimination results from the upregulation of enzymes that intercept and degrade the insecticide before it reaches the target site. (**B**) Target site modification results from the changing of a target site such that it cannot interact with the insecticide. (**C**) Cuticle thickening results from an increase in cuticular thickness and a decrease in cuticular permeability, thereby preventing an insecticide from entering the body. (**D**) Behavioral avoidance results from the changing of behavior such that the insect avoids encountering an insecticide.

When first used as insecticides, axonic and synaptic poisons were highly effective against mosquitoes. However, their continued use has exerted selective pressure on their neurophysiological targets and resistance against these poisons has both evolved and spread [11,29,30,31,32,33]. This resistance most often evolves through one or more of four different types of adaptations: (i) increased metabolic elimination or sequestration of the insecticide, (ii) a change in target site structure that prevents insecticide binding, (iii) a change in the insect cuticle to limit the entry of the insecticide into the body, or (iv) a behavioral modification that results in the avoidance of the insecticide [11,34,35] (Figure 2). Metabolic elimination of the toxin and target site modification that prevents the toxin from interfering with voltage gated sodium channels are two common strategies evolved by mosquitoes to resist insecticides [6,11,32,36,37].

**Figure 3:**
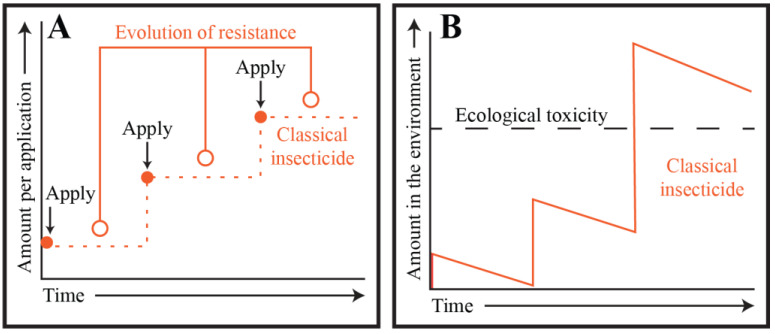
**A model of accumulation and resistance of insecticides, known as the pesticide treadmill.** (**A**) Resistance evolves (open circles) against classical insecticides following their repeated application (closed circles) because of selective pressure on their highly specific neurological targets. Therefore, to achieve the same level of insect control, higher dosages are progressively applied as the populations gain resistance. (**B**) Classical insecticides persist in the environment and require increased dosage application to manage resistant populations. Therefore, classical insecticides accumulate over time and eventually lead to ecological damage.

According to the 2021 World Malaria Report, 78 of the 88 malaria endemic countries that were surveyed reported mosquitoes that are resistant to neurotoxic insecticides [6]. As resistance has spread, some insecticides have been abandoned because they have lost their efficacy. Others have been applied in a prescribed rotational sequence, at significantly higher dosages, or as part of complex pesticide mixtures—a concept known as the pesticide treadmill [38,39] (Figure 3A). Although, the rotational application of insecticides delays the selection of resistant insects, the environmental accumulation of residual amounts of multiple insecticides selects for insects that are resistant to a broad range of insecticides [40,41,42,43]. In some cases, this renders insects resistant to insecticides that have never been applied in their environment, dramatically limiting the arsenal of viable insecticides [35,40,44,45,46]. As the development of cross resistance becomes increasingly prevalent and the list of viable insecticides dwindles, so too does our ability to choose insecticide regimens that are minimally disruptive to the environment [47].

## 3. Targeting Mosquito Adults: The Ecological Cost of Classical Chemical Insecticides

Once resistance evolves and becomes widespread, higher dosages are needed to reach the same level of insect control. The excessive use of insecticides not only selects for resistant insect populations but also leads to environmental accumulation and harm to the surrounding ecosystem (Figure 3B). This ecological damage—such as the eggshell thinning of birds [48,49] or the developmental defects of amphibians [50,51,52,53,54]—results from the chronic exposure that non-target vertebrate and invertebrate animals face following the accumulation and persistence of insecticides in the environment [55,56,57,58]. At high enough levels, these insecticides also disrupt neurophysiological processes in humans [59,60,61], and the toxicity of multiple insecticides can synergize to cause negative consequences that are often difficult to predict [62,63,64,65,66,67].

The environmental lifetime of an insecticide, or the duration of time that a pesticide persists in the environment following its application, is dictated by the mechanism of degradation as well as the surrounding biotic and abiotic factors that modulate the rate of degradation [68]. Classical insecticides are predominantly degraded by microbes through metabolic processes that can be slow [68]. This leaves insecticides in the ecosystem for years following their application [68,69,70,71,72,73]. Moreover, this extended persistence feeds back into the pesticide treadmill; chronic exposure to an insecticide promotes the development of resistance, which then requires higher dosages and rotational applications to control insect populations. Therefore, to escape this treadmill, insecticides with reliably brief environmental lifetimes are needed. With this in mind, non-chemical means of control, such as entomopathogenic fungi, the sterile insect technique, *Wolbachia*, and gene drives, have all been developed and showed to be effective in controlling insect populations or reducing disease transmission [74,75,76,77,78]. These approaches show promise but are limited by environmental factors and practical constraints; for example, *Wolbachia* infection varies with temperature and diet [78], and determining efficient methods of storage, preservation, formulation, and application of entomopathogenic fungal pesticides is laborious [79]. A complementary way to control mosquito populations that escapes some of these constraints involves targeting an earlier life stage of the mosquito: the larva.

## 4. Targeting Mosquito Larvae: A Limited Arsenal of Insecticides

**Figure 4:**
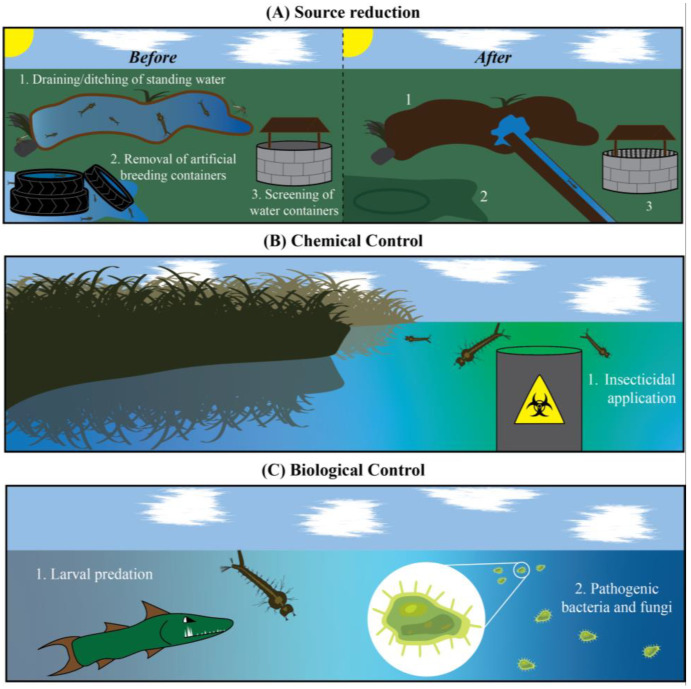
**Controlling larval populations relies on three general strategies: source reduction, chemical control, and biological control.** (**A**) Source reduction decreases mosquito access to standing water, preventing both oviposition and larval development. (**B**) Chemical control kills larvae via toxic insecticides. (**C**) Biological control introduces an organism, such as a larval predator, bacteria, or fungi, that kills larvae.

Mosquito populations and disease transmission can be reduced by targeting the immature larval stage via source reduction, larvicidal chemicals, and biological control (Figure 4). Reducing the larval population has an intragenerational effect that lowers the density of vector-competent adults while at the same time having a transgenerational effect that curtails reproduction, and by extension, the next generation of mosquitoes. Prior to the advent of synthetic insecticides and biological control agents, the primary form of mosquito control was source reduction, or the modification of habitats to reduce oviposition sites and opportunities for larval development. While effective in reducing mosquito density, the maintenance, cost, and unintended effects—especially when impounding or ditching—often makes these approaches prohibitive.

Chemical larvicides can be subdivided into two functional subclasses: growth regulators and synthetic neurotoxins. Growth regulators interfere with metamorphosis by either inhibiting chitin synthesis or by serving as juvenile hormone analogs that disrupt the hormonal signaling that drives ecdysis [4,80]. Insect growth regulators do not adversely affect vertebrate animals, but their insecticidal activity is restricted to the time around molting. Synthetic neurotoxins target axons and synapses, and like those used against adults, include chlorinated hydrocarbons, organophosphates, neonicotinoids, and pyrethroids [4,28,81]. These insecticides tend to persist in the environment long after their application, which negatively affects the development of species as disparate as invertebrates, amphibians, reptiles and birds [54,58,61,82,83]. Natural products like plant oils can also kill larvae, but this requires intensive purification, and their effectiveness is mixed [84,85]. For that reason, and because of the evolution of resistance, many larvicidal chemicals have been removed from the market or used sparingly [12,81].

Biological control relies on invertebrates and fish that are natural larval predators, or larvicidal bacteria that interfere with intestinal homeostasis. Larval predators are undoubtedly effective, but this control strategy is painstakingly difficult to maintain and impractical for the control mosquitoes that breed in small bodies of water like artificial containers. Larvicidal bacteria, such as the spinosyn-producing *Saccaropolyspora spinosa*, the Bin-producing *Lysinibacillus sphaericus*, and the Cry-producing *Bacillus thuringiensis israelensis* (*Bti*), are unique in that they selectively disrupt intestinal homeostasis in mosquitoes and related Nematocera [4,86,87,88,89,90]. Due to its high specificity and high larvicidal efficiency, *Bti* and its Cry toxins have become the gold standard for the biological control of mosquito larvae.

Although *Bti*-based insecticides have been very successful at suppressing mosquito populations, the evolution of resistance, which albeit at the moment is rare, may threaten their efficacy [86,91,92,93,94,95]. Resistance to *Bti* has emerged in mosquito populations that have been intentionally treated in the laboratory with sub-lethal dosages of *Bti* over time, and has been detected in a survey of natural mosquito populations in Upstate New York [93,96,97,98]. In mosquitoes, the mechanism of *Bti* resistance remains poorly understood, but in non-mosquito invertebrates it has evolved via target site modification of midgut receptors and metabolic sequestration that inactivates the toxins [92]. It is possible that *Bti* resistance is rare because it is accompanied by a fitness cost: *Bti* resistant mosquitoes develop more slowly, produce fewer eggs, and fewer eggs are viable [97,99]. Nevertheless, considering *Bti* as an infallible larvicide—much like DDT was once considered an infallible adulticide—would restrict our ability to control mosquito populations if resistant populations were to emerge more commonly. Moreover, there are epidemiological concerns regarding *Bti* use because a sub-lethal exposure to *Bti* enhances dengue susceptibility in *Aedes aegypti* [100]. Overall, current methods for larval control are subject to the development of resistance, limited larvicidal activity, logistical hurdles, unintended consequences to non-target organisms, or resource limitations.

## 5. A Case for Photosensitive Insecticides as Mosquito Larvicides

**Figure 5:**
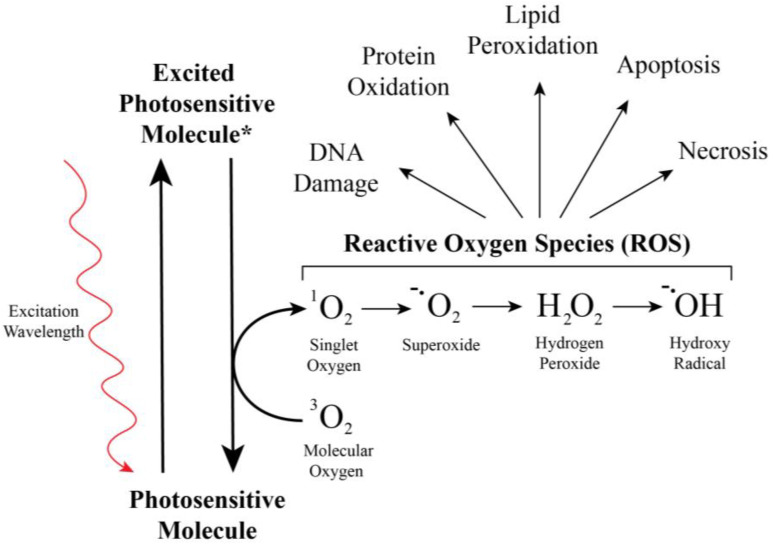
**Photosensitive molecules are activated by light to produce reactive oxygen species.** Reactive oxygen species produced by the photoactivation (asterisk) of a photosensitive insecticide irreversibly damage biomolecules, harming the organism in numerous ways.

An attractive addition to our larvicidal arsenal is chemicals that function via a broad molecular mechanism yet have minimal negative consequences against non-target organisms. Light activated water soluble larvicides—which we call photosensitive insecticides or PSIs—fit this mold. These molecules are activated by light via photosensitization to generate toxic and short-lived reactive oxygen species, or ROS [101,102,103,104,105]. Photosensitization occurs when a process known as intersystem crossing—or the conversion of electron spin states—extends the excited state of a molecule that has absorbed light. Following intersystem crossing, the excited molecule transfers its energy to molecular oxygen to create ROS that harm cells via DNA damage, protein oxidation, lipid peroxidation, necrosis, and inducing apoptosis (Figure 5). This energy transfer is a cyclic process and PSIs can catalyze the production of ROS continuously whilst irradiated until the PSI is ultimately photodegraded. Although many different types of molecules can undergo photosensitization, several molecular classes, such as thiazines, porphyrins, xanthenes, fluoresceins, anthracenes and beta-di-ketones, produce ROS in sufficient quantities to be toxic against insects [106,107,108,109,110,111,112]. In the past, these molecules have been referred to as photosensitive insecticides, photodynamic pesticides, photoactive pesticides, light-activated pesticides or photosensitizing agents.

**Figure 6:**
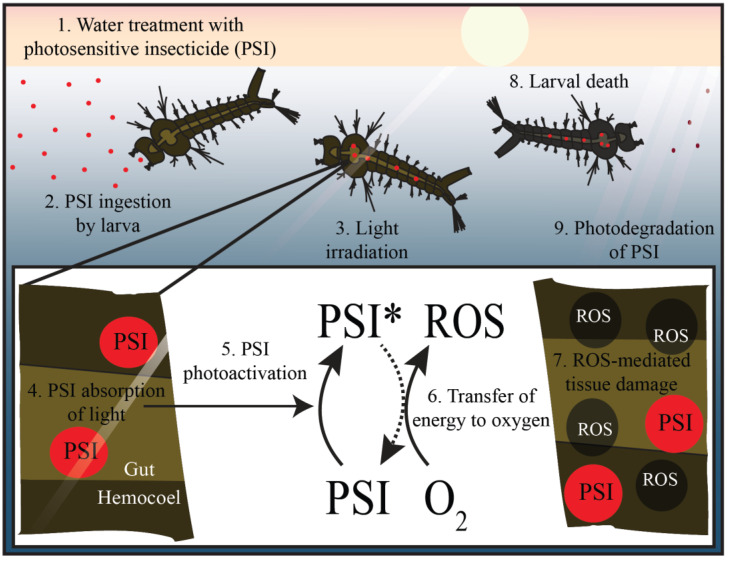
**Photosensitive insecticides (PSIs) are ingested by larvae and kill them via oxidative damage.** Photosensitive insecticides are applied to bodies of water (1), and once ingested by larvae (2), the PSIs are activated by natural light (asterisk) to produce reactive oxygen species (ROS; 3–6). These ROS irreversibly damage macromolecules in their vicinity, killing the larvae (7, 8). Any PSI that is not consumed is degraded by sunlight (9).

For PSIs to be toxic, they need to be photoactivated inside an organism. When this occurs, the ROS that are produced react indiscriminately with proximal organic macromolecules and irreversibly damage them. If sufficient damage ensues, the organism dies (Figure 6) [107]. If generated outside of the organism, ROS cannot diffuse into or across tissue due to their brief half-life which is on the scale of microseconds [113]. Unlike other broadly toxic chemicals, PSIs are only toxic to organisms that (i) readily ingest PSIs and (ii) are sufficiently small and translucent for light to penetrate the body and activate the PSI. Although this means that the toxicity of PSIs is not limited to mosquito larvae, PSIs do not harm opaque or large organisms. For example, at a dosage lethal to mosquito larvae, the PSI curcumin does not damage translucent zebrafish even after significant ingestion [114].

The capacity for PSIs to effectively kill mosquito larvae is an exciting prospect when considering the potential use of PSIs against mosquito populations that are resistant to classical insecticides. Due to the lack of a specific target site, PSIs are predicted to retain their toxicity in mosquito populations that are resistant to other larvicides. By not targeting a specific physiological process, neither target site modification, behavioral adaptation, or cuticle thickening would protect larvae from PSIs. Only the upregulation of detoxification enzymes could conceivably provide protection, but the fast-acting and highly transient nature of ROS provides only a brief window for enzymes to mitigate the damage [115]. Therefore, resistant populations are unlikely to emerge.

**Figure 7:**
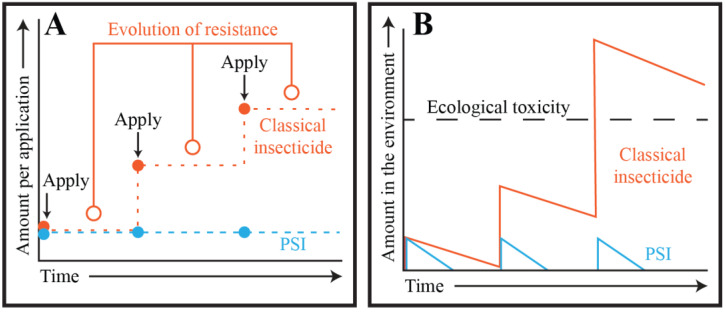
**Photosensitive insecticides avoid the pesticide treadmill and environmental accumulation.** (**A**) Resistance evolves (open orange circles) against classical insecticides following their repeated application (closed orange circles) because of selective pressure on their highly specific neurological targets whereas the evolution of resistance against PSIs following their repeated application (closed blue circles) is unlikely because of their broad mechanism of action that relies on oxidative damage. Therefore, to achieve the same level of insect control with classical insecticides, higher dosages are progressively applied as the populations gain resistance, whereas this is unnecessary for PSIs. (**B**) Classical insecticides persist longer in the environment and require increased dosage application to manage resistant populations. Therefore, classical insecticides accumulate over time and eventually lead to environmental damage, whereas PSIs do not.

**Figure 8:**
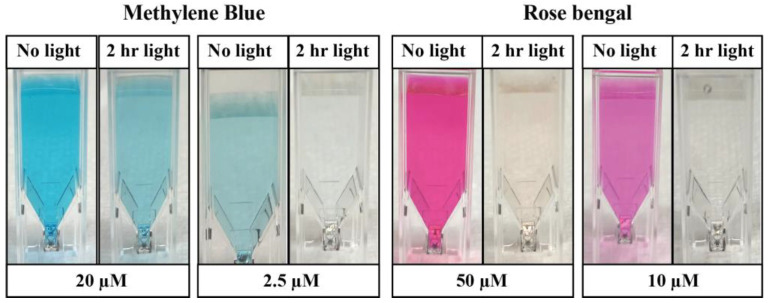
**Photosensitive insecticides are broken down by photodegradation.** Images of various concentrations of the PSIs, methylene blue and rose bengal, that have been maintained in the dark or exposed to 5000 Lumens of LED light for 2 h. Photodegradation is evident by the increased clarity and translucency following light irradiation.

PSIs also do not carry one of the burdens of classical chemical insecticides: the pesticide treadmill and environmental accumulation (Figure 7). The environmental lifetime of PSIs is very brief. Rather than relying on microbial degradation, PSIs undergo photodegradation—an inevitable outcome of its photosensitivity. When activated by light, the ROS generated by a PSI readily react with the parent PSI, which degrades the molecule and prevents their environmental accumulation [116,117,118,119]. In fact, when irradiated at naturally occurring light intensities, PSIs degrade in just a few hours (Figure 8); this rate can be further expedited using nanoparticle catalysts that increase the rate of ROS production [116,118,120,121,122]. Importantly, PSI photodegradation yields naturally occurring nutrients such as CO_2_, NH_4_^+^, NO_3_^−^, and SO_4_^2−^, all of which are sequestered by plants and used for their metabolic processes [116,123]. By relying on photosensitization and photodegradation, PSIs are highly potent larvicides that rapidly degrade in the environment.

Given the potential to control mosquito populations that are resistant to classical insecticides, together with the brief environmental lifetime of PSIs and the unlikely prospect that resistance against them emerges, PSIs offer a unique and promising escape from the pesticide treadmill. Even if resistance were to evolve, PSIs would provide a welcome respite from classical insecticide practices, giving the ecosystem the time needed to recover from the effects of classical insecticide accumulation.

## 6. Photosensitive Insecticides: Practical Outcomes and Outstanding Questions

Mosquito larvae are small, translucent, and live in an aquatic habitat, making them ideal PSI targets. The thiazine, methylene blue, and the beta-di-ketone, curcumin, are lethal to *Aedes aegypti* larvae under laboratory and simulated field conditions [110,111,124,125]. The fluorescein derivative, rose bengal, kills both *Anopheles gambiae* and *Aedes aegypti* in laboratory conditions, and *Culex quinquefasciatus* in both laboratory and field conditions [126]. Beyond mosquito larvae, the porphyrin, TMPYP, kills larval *Chaoborus sp.* (Diptera: Nematocera), and the xanthene derivative, phyloxine-b, kills adult *Anastrepha ludens* (Diptera: Tephritidae), *Melanoplus sanguinipes* (Orthoptera: Acrididae) and *Schistocerca americana* (Orthoptera: Acrididae) [127,128,129].

Importantly, PSIs are considered non-toxic to humans, and the opacity of the skin prevents their photoactivation. In fact, methylene blue was an early drug used to treat human malaria [130], and is used as a vasopressor and to treat shock [131,132]. Rose bengal is being investigated as a treatment against infectious keratitis and malignant melanoma [133,134,135]. Photosensitive molecules have also been used in phototherapy to target cancerous cells and are non-toxic without irradiation [136,137,138,139,140,141]. Although the effectiveness of PSIs as therapeutic drugs is uncertain, the side effects associated with ingestion by humans were not reported or minor. Methylene blue and rose bengal are usually thought of as deeply colored histological dyes; however, the concentrations used for histology are orders of magnitude greater than when used as a PSI.

Despite the predicted safety of PSIs to humans, some concerns and outstanding questions remain surrounding their ecological impact. Like classical chemical insecticides, PSIs are likely to kill non-target arthropods—so long as they are small and translucent—in addition to mosquito larvae. Therefore, experiments regarding the long-term effects of PSIs on biodiversity are needed. Furthermore, despite the brief environmental lifetime of PSIs due to photodegradation, their breakdown requires sunlight. Therefore, PSIs may accumulate in dark areas [68]. This limitation is minor because any PSI that persists in dark areas would be in its inactive state, so it would not cause ecological damage. However, this also means that PSIs would be ineffective in underground storm drains and catch basins, which in urban settings are larval breeding sites that are largely devoid of sunlight. A more realistic limitation of PSIs is that by degrading so quickly when photoactivated, frequent re-applications may be necessary to continuously manage insect populations. Innovations in insecticide delivery—such as yeast encapsulation of essential oil larvicides, photosensitive insecticidal paint emulsions, or water soluble capsules that release PSIs during rainfall when larval hatching is stimulated—should increase the stability and ease of delivery of these insecticides [142,143,144,145]. However, the application of PSIs will probably always be more effort-intensive compared with classical insecticides. This limitation will likely prevent the replacement of classical insecticides with PSIs, although their (i) effectiveness, (ii) much lower probability that resistance evolves and (iii) low cost may make their use well worth the additional effort. The application of PSIs to small bodies of water near dwellings should significantly reduce mosquito populations near human habitation, thereby decreasing the burden of mosquito-borne disease.

Additionally, the unlikelihood that mosquito larvae evolve resistance against PSIs needs to be empirically tested. Although the mechanism of PSI toxicity suggests that resistance is improbable, one study in adult house flies fed erythrosine B reported a shift toward resistance over time [146]. However, it is unclear whether toxicity in that study—and therefore the mechanism of resistance—is linked to phototoxicity, especially because this species is larger and significantly less translucent than mosquito larvae. Moreover, studies conducted in bacteria suggest that resistance is unlikely to evolve. PSIs have antibacterial activity that is also based on ROS production via photoactivation, yet attempts in the laboratory to select for resistant bacterial populations have failed [115,147,148,149,150,151,152,153]. Only when bacteria were genetically engineered to be pigmented did resistance to PSIs increase, presumably because their opacity prevented light-based activation of the photosensitive molecules [154]. Whether this pigmentation phenotype can naturally evolve in insects is unknown. However, for mosquitoes this may be an ineffective resistance strategy because the larvae of many insects rely on their level of pigmentation to hide from predators [155,156]. Therefore, because of the unlikely probability that resistance emerges, PSIs should not need to be applied in increasing doses over time, or used in a pesticide treadmill, for them to remain effective (Figure 7).

It is unclear how the toxicity of PSIs extends across the entire mosquito lineage. PSIs are toxic to larvae in both major subfamilies of mosquitoes—Anophelinae and Culicinae—but differences in larval size and translucency in both lineages means that PSI potency is likely to vary between the thousands of mosquito species that have been described. The experiments that have assessed PSI toxicity in a handful of mosquito species have used different (i) PSIs, (ii) dosages, (iii) exposure durations, (iv) irradiation sources and (v) irradiation durations, which makes it challenging to compare the toxicity of PSIs between mosquito species. Furthermore, it is difficult to evaluate the differences in larvicidal efficiency between PSIs, even when targeting the same mosquito species. Therefore, experiments that elucidate the potency of different PSIs across the mosquito lineage are needed to optimize the efficacy of PSIs.

Finally, as with any form of mosquito control, if PSIs were to be deployed it would be inevitable that some larvae are exposed to a sublethal dose. The larvae that survive such an exposure may suffer negative life history consequences, such as decreased longevity, reproductive output or vectorial capacity; oxidative stress in adults reduces fecundity and longevity, and reduces insecticide detoxification capacity [157,158]. For example, *A. aegypti* larvae that survive exposure to curcumin develop more slowly, exhibit an altered sex ratio, and have reduced adult longevity [111]. However, a sub-lethal PSI exposure could also result in hormesis, where low-level exposure to a harmful substance yields a benefit [38,159]. If this were to occur in a mosquito before an infection is contracted, the larva would eclose into an adult that has a stronger redox response, thereby better tolerating a low intensity infection and surviving longer. Therefore, the effects of a sub-lethal PSI exposure on life history traits must be explored.

## 7. Conclusions

Classical chemical insecticides reduce mosquito populations and curtail the transmission of disease. However, in regions of robust application, this benefit comes with a reduction in efficacy because of the evolution of resistance and a significant ecological burden because of environmental accumulation. PSIs are a promising addition to our arsenal of insecticides because (i) they target the larval stage of the mosquito which offers both intragenerational and transgenerational vector control, (ii) the mechanism of action is non-specific and therefore unlikely to select for resistance, (iii) their self-degradation prevents their environmental accumulation, (iv) the light-activated mechanism is harmless to opaque organisms such as humans, and (v) they are readily available and inexpensive. Therefore, with the growing concerns surrounding the use of classical insecticides, PSIs are a promising alternative for controlling mosquito populations in an environmentally sustainable manner.

## Data Availability

No new data were created or analyzed in this study. Data sharing is not applicable to this article.

## Supplementary Materials

The following supporting information can be downloaded at: https://www.mdpi.com/article/10.3390/insects13121093/s1, Figure S1: Global use of insecticides for vector control between 2010 and 2019, divided by the type of intervention.
