# Supplementary material for: Chemical Control of Mosquitoes and the Pesticide Treadmill: A Case for Photosensitive Insecticides as Larvicides
Source: Insects. 2022 Nov 28;13(12):1093. doi: 10.3390/insects13121093 (PMC9783766; doi:10.3390/insects13121093)
Supplement: Supplementary file 1 [file insects-13-01093-s001.zip › insects-2013222-supplementary.pdf]

insects

MDPI

Review – Supplementary Material

Chemical Control of Mosquitoes and the Pesticide Treadmill: a Case for Photosensitive Insecticides as Larvicides

Cole J. Meier [1], Matthew F. Rouhier [2] and Julián F. Hillyer [1,*]

[1] Department of Biological Sciences, Vanderbilt University, Nashville, TN 37235, USA
[2] Department of Chemistry, Kenyon College, Gambier, OH 43022, USA
* Correspondence: julian.hillyer@vanderbilt.edu

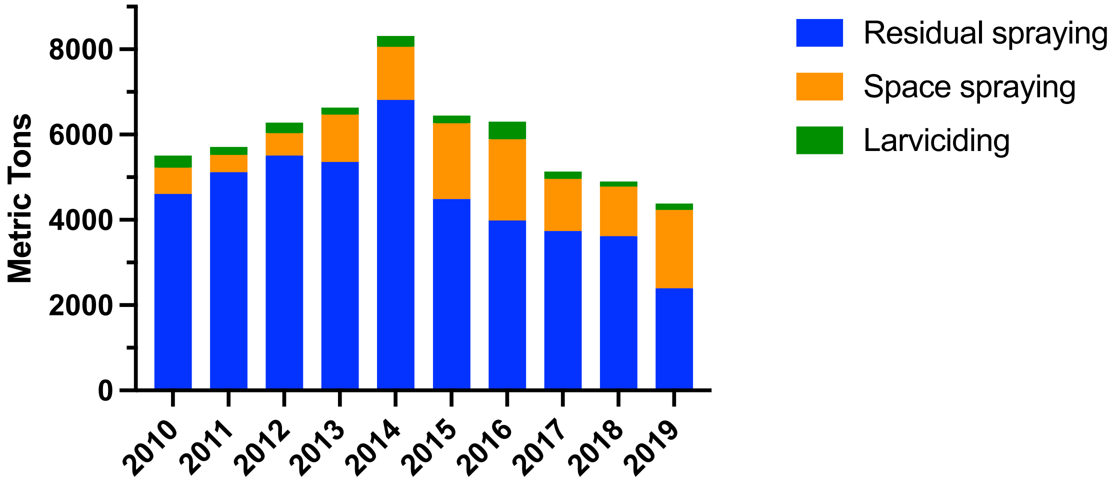

Figure S1. Global use of insecticides for vector control between 2010 and 2019, divided by the type of intervention. Adulticidal residual spraying includes organochlorines, organophosphates, carbamates, pyrethroids and neonicotinoids. Adulticidal space spraying includes organophosphates, carbamates, pyrethroids and neonicotinoids. Larviciding includes organophosphates, pyrethroids, bacterial larvicides, insect growth regulators and spinosyns. Data were extracted from Table 7 of the World Health Organization’s (WHO) 2021 report titled *Global insecticide use for vector-borne disease control: a 10-year assessment (2010–2019)*, 6th edition.
